# cGMP Signaling in Photoreceptor Degeneration

**DOI:** 10.3390/ijms241311200

**Published:** 2023-07-07

**Authors:** Shujuan Li, Hongwei Ma, Fan Yang, Xiqin Ding

**Affiliations:** Department of Cell Biology, College of Medicine, University of Oklahoma Health Sciences Center, Oklahoma City, OK 73104, USA; shujuan-li@ouhsc.edu (S.L.); hongwei-ma@ouhsc.edu (H.M.); fanyang8109@gmail.com (F.Y.)

**Keywords:** cGMP, PKG, PDE6, CNG channel, photoreceptor degeneration, retina

## Abstract

Photoreceptors in the retina are highly specialized neurons with photosensitive molecules in the outer segment that transform light into chemical and electrical signals, and these signals are ultimately relayed to the visual cortex in the brain to form vision. Photoreceptors are composed of rods and cones. Rods are responsible for dim light vision, whereas cones are responsible for bright light, color vision, and visual acuity. Photoreceptors undergo progressive degeneration over time in many hereditary and age-related retinal diseases. Despite the remarkable heterogeneity of disease-causing genes, environmental factors, and pathogenesis, the progressive death of rod and cone photoreceptors ultimately leads to loss of vision/blindness. There are currently no treatments available for retinal degeneration. Cyclic guanosine 3′, 5′-monophosphate (cGMP) plays a pivotal role in phototransduction. cGMP governs the cyclic nucleotide-gated (CNG) channels on the plasma membrane of the photoreceptor outer segments, thereby regulating membrane potential and signal transmission. By gating the CNG channels, cGMP regulates cellular Ca^2+^ homeostasis and signal transduction. As a second messenger, cGMP activates the cGMP-dependent protein kinase G (PKG), which regulates numerous targets/cellular events. The dysregulation of cGMP signaling is observed in varieties of photoreceptor/retinal degenerative diseases. Abnormally elevated cGMP signaling interferes with various cellular events, which ultimately leads to photoreceptor degeneration. In line with this, strategies to reduce cellular cGMP signaling result in photoreceptor protection in mouse models of retinal degeneration. The potential mechanisms underlying cGMP signaling-induced photoreceptor degeneration involve the activation of PKG and impaired Ca^2+^ homeostasis/Ca^2+^ overload, resulting from overactivation of the CNG channels, as well as the subsequent activation of the downstream cellular stress/death pathways. Thus, targeting the cellular cGMP/PKG signaling and the Ca^2+^-regulating pathways represents a significant strategy for photoreceptor protection in retinal degenerative diseases.

## 1. Introduction

The human retina is comprised of light-responsive neuronal cells known as photoreceptors. Light stimulation initiates phototransduction in photoreceptors, which is conveyed to secondary neurons, ganglion cells, and, ultimately, to the visual cortex in the brain to form vision. There are two types of photoreceptors: rods and cones. Rods respond to dim light and enable vision at night, whereas cones react to bright light and are responsible for daylight vision, color vision, and visual acuity [1,2,3,4]. Photoreceptors degenerate in many hereditary retinal diseases, including retinitis pigmentosa (RP: a progressive degeneration of rods leading to blindness), Leber congenital amaurosis (LCA: many LCA forms involve severe dystrophy of photoreceptors), achromatopsia (rod monochromatism, color blindness), cone-rod dystrophy (CRD), and age-related retinal degeneration, such as age-related macular degeneration (AMD). Despite the remarkable heterogeneity of disease-causing genes, environmental factors, and pathogenesis, the progressive death of rod and cone photoreceptors ultimately leads to the loss of vision/blindness.

**Figure 1:**
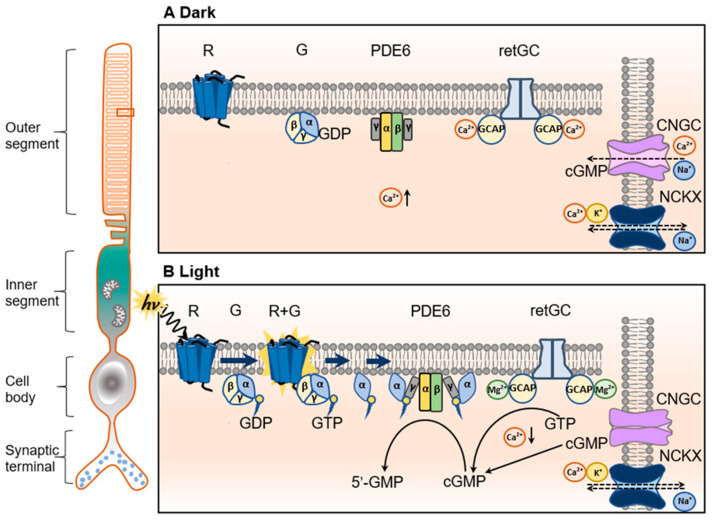
The phototransduction and regulation of cGMP signaling in a representative rod photoreceptor. (**A**) In the dark, cGMP, at a high intracellular level, binds to the CNG channel to keep it open, which allows Ca^2+^ and Na^+^ influx and subsequently elevates [Ca^2+^]_i_. The Ca^2+^-bound GCAP binds and inactivates RetGC at high [Ca^2+^]_i_, exerting the negative feedback control to cGMP synthesis. In the meantime, Ca^2+^ ions are steadily extruded via the Na^+^/Ca^2+^-K^+^ exchanger (NCKX) in the outer segment and via Ca^2+^-ATPase from the inner segment (not shown), thus maintaining the constant free Ca^2+^ levels. (**B**) In light stimulation, photon (*hv*) absorption leads to a conformational rearrangement of the rhodopsin (R) protein, which activates multiple copies of the heterotrimeric G protein transducin (G), causing the exchange of GTP for GDP on its α subunit. The activated Gα subunit (GTP-bound) binds to the γ-subunit of PDE6, relieving the inhibitory constraint and leading to the catalytic acceleration of cGMP hydrolysis. Reduced free cGMP levels lead to the closure of the CNG channel, reduction in the Ca^2+^ influx, and the subsequent reduction in [Ca^2+^]_i_. Along with the continued activity of NCKX, the membrane is hyperpolarized, the electro-chemical signal is transmitted, and the synaptic glutamate release is altered. As a result of reduced [Ca^2+^]_i_, the Mg^2+^-bound/Ca^2+^-free GCAP binds to RetGC and activates cGMP synthesis, forming a feedback loop to open the CNG channel over again (R, rhodopsin; G, transducin; CNGC, CNG channel; NCKX, Na^+^/Ca^2+^-K^+^ exchanger; modified from Leskov et al. [7] and Tolone et al. [13].

Cyclic guanosine 3′, 5′-monophosphate (cGMP) acts as a second messenger molecule in almost all cell types by activating the cGMP-dependent protein kinase G (PKG) and regulating various cellular activities. In photoreceptors, besides acting as a second messenger, cGMP plays a pivotal role in phototransduction by governing the cyclic nucleotide-gated (CNG) channels. In darkness, CNG channels are kept open by the binding of cGMP, maintaining a steady Ca^2+^ and Na^+^ influx. Upon light stimulation, the light-sensing protein rhodopsin/opsin undergoes a conformational rearrangement, which activates the heterotrimeric G protein transducin. Consequently, the α subunit of transducin activates phosphodiesterase 6 (PDE6), leading to the catalytic acceleration of cGMP hydrolysis, closure of the channels, and the hyperpolarization of the cell [5,6,7,8,9] (Figure 1). Phototransduction follows the same cascade in both rods and cones, even though the components involved in the cascade vary in the two types of the photoreceptors. For example, the rod CNG channel is composed of CNGA1 and CNGB1 subunits, whereas the cone CNG channel is composed of CNGA3 and CNGB3 [6,10]. The rod PDE6 is composed of PDE6α, PDE6β, and PDE6γ subunits, whereas the cone PDE6 is composed of the PDE6α’ and PDE6γ’ subunits [11,12].

Among the various cellular alterations that occur in photoreceptor degeneration and the potential causative factors, dysregulation of cGMP signaling appears to be one of the leading features. The dysregulation of cGMP signaling is associated with the pathogenesis of RP, LCA, achromatopsia, and CRD [14], and manipulations that reduce the cellular cGMP level result in photoreceptor protection in animal models of retinal degeneration. Here, we review the regulation of cGMP signaling in photoreceptors, the dysregulation of cGMP signaling in photoreceptor degeneration, the experimental evidence supporting the contribution of the elevated cGMP signaling to photoreceptor degeneration, and the potential mechanisms underlying cGMP signaling-induced photoreceptor degeneration. A better understanding of cGMP signaling in photoreceptor degeneration will help the development of therapeutic interventions targeting this signaling pathway for photoreceptor protection.

## 2. Regulation of Cellular cGMP Level in Photoreceptors

The cellular level of cGMP is highly regulated in photoreceptors. The biosynthesis of cGMP is catalyzed by the retinal guanylyl cyclases (RetGCs) from the guanosine triphosphate (GTP), and its degradation is achieved by PDE6, which specifically hydrolyzes the 3′5′ cyclic phosphate bond [15] (Figure 1). The biosynthesis of cGMP is tightly regulated by guanylate cyclase activator proteins (GCAPs) [16,17]. There are four basic elements regulating cellular cGMP levels in photoreceptors, e.g., PDE6, RetGC, GCAP, and intracellular Ca^2+^ level ([Ca^2+^]_i_). In addition, the functionality of the photoreceptor CNG channels affects the cellular cGMP levels indirectly via [Ca^2+^]_i_.

### 2.1. The Ca^2+^/Mg^2+^-GCAP/RetGC Complex

The production of cGMP in photoreceptors is catalyzed by RetGCs. There are two subtypes of RetGCs: RetGC1 and RetGC2. RetGC1 is encoded by *GUCY2D* (*Gucy2e* in mouse), and RetGC2 is encoded by *GUCY2F* (*Gucy2f* in mouse) [16,18]. RetGC1 is the main isozyme of the cyclases, expressed in both rods and cones, whereas RetGC2 is primarily expressed in rods, and it is nearly undetectable in cones [19,20]. RetGC1 and RetGC2 act as homodimers and share similar domain structures with other membrane-bound guanylyl cyclases [21,22,23]. They both contain an extracellular ligand-binding domain, a dimerization domain, intracellular protein kinase homology domains, and cyclase catalytic domains. Although the three-dimensional structure of RetGC has not been determined, its working pattern has been demonstrated. The two opposing subunits of the RetGC homodimer bind to two Mg^2+^-GTP substrate molecules, simultaneously with their catalytic domains, creating a single active site to convert GTP to cGMP [24,25]. RetGC1 is responsible for over 70% of the cGMP production in photoreceptors, whereas the RetGC2 isozyme serves as the ancillary component, producing less than 30 percent of the total cGMP [20].

The activity of RetGC is highly regulated by GCAP which binds to RetGC to form a RetGC/GCAP complex (Figure 1). There are two subtypes of GCAPs: GCAP1 and GCAP2. GCAP1 virtually regulates RetGC1 only, whereas GCAP2 activates both RetGC1 and RetGC2 [26,27,28]. The structure of the RetGC/GCAP complex remains less clear. It is known that the activity of RetGC, in the complex, is correlated strongly with the Ca^2+^ or Mg^2+^ ligands of GCAP. GCAPs are recoverin-like Ca^2+^ sensor proteins containing a helix–loop–helix metal-binding structure [29,30]. When the helix–loop–helix metal-binding domains are occupied by Ca^2+^, the complex inhibits RetGC and reduces cGMP synthesis. When the metal-binding domains are occupied by Mg^2+^, the complex activates RetGC and stimulates cGMP synthesis [17,31,32]. In dark-adapted photoreceptors, where [Ca^2+^]_i_ is high, the Ca^2+^-bound GCAPs inactivate RetGC and inhibit cGMP synthesis (Figure 1). In light-activated photoreceptors, where [Ca^2+^]_i_ is low, the metal-binding domains of GCAPs are replaced by Mg^2+^ (Mg^2+^-bound/Ca^2+^-free form), and the Mg^2+^-bound GCAPs activate RetGCs and stimulate cGMP synthesis (Figure 1).

In addition, the activity of RetGCs is regulated by the retinal degeneration protein 3 (RD3). RD3 is a 23-kDa monomer with an elongated four-helix bundle [33]. The in vitro and in vivo experiments show that the main role of RD3 is to suppress the aberrant activation of RetGC by GCAPs in the inner segment [34]. It is required for the stability and ciliary trafficking of RetGCs, and it suppresses the activity of the enzymes in the inner segment before trafficking to the outer segment [35].

### 2.2. PDE6

PDE6 is the primary regulator of intracellular cGMP concentration in rod and cone photoreceptors (Figure 1). PDE enzymes are a superfamily containing 11 members (PDE1-11) in humans, which are structurally related but consist of multiple genes and function distinctly [36]. PDE6 belongs to 3′,5′-cyclic nucleotide PDEs that catalyze the hydrolysis of the phosphodiester bond of cyclic nucleotides [principally, cGMP and cyclic adenosine monophosphate (cAMP)] [37,38]. There are rod PDE6 complexes and cone PDE6 complexes. Rod PDE6 consists of two catalytic subunits—PDE6α and PDE6β—and two identical inhibitory PDE6γ subunits, whereas cone PDE6 is composed of two identical PDE6α’ catalytic subunits and two identical cone-specific PDE6γ’ inhibitory subunits [15,39]. In dark-adapted photoreceptors, the activity of the catalytic subunits is inhibited by the inhibitory γ-subunit at the entrance of the active site [40]. Upon light stimulation, the photopigment rhodopsin/opsins undergo a conformational change, which activates transducin, and the α subunit of transducin binds to the γ-subunit of PDE6, relieving the inhibitory constraint and activating the enzyme (Figure 1).

### 2.3. CNG Channel

In addition to regulation by the Ca^2+^/Mg^2+^-GCAP/RetGC complex and PDE6, cellular cGMP level is indirectly controlled by photoreceptor CNG channels via [Ca^2+^]_i_ (Figure 1). Photoreceptor CNG channels are heterotetrameric complexes located on the plasma membranes of the outer segments. They belong to the superfamily of pore–loop cation channels [6,10]. The CNG channel family is comprised of six homologous members, which are classified as structurally similar A subunits (CNGA1-4) and B subunits (CNGB1 and CNGB3), assembled into distinct cell types/specific combinations of heterotetramers [41]. The rod CNG channel is a heterotetramer consisting of three CNGA1 and one CNGB1 [42,43]. Similarly, the cone CNG channel is a heterotetramer consisting of three CNGA3 and one CNGB3 [44,45]. CNG channels are strictly ligand-gated ion channels because the channel-opening state requires the binding of cGMP or cAMP to their cyclic nucleotide-binding domain [46]. Opening the channels allows for an influx of Na^+^ and Ca^2+^ ions. In darkness, Ca^2+^ ions carry about 20% of the ionic current flowing through the channel in rods, whereas this fraction is about 35% in cones [47]. The resulting influx of Na^+^ and Ca^2+^ (so called “dark current”) keeps the plasma membrane depolarized and promotes the synaptic glutamate release. As a feedback regulatory mechanism, the elevated [Ca^2+^]_i_ inhibits cGMP synthesis via the GCAP/RetGC complex. The elevated [Ca^2+^]_i_ also slightly reduces the affinity of cGMP to the channel [48]. Light triggers hydrolysis of cGMP, leading to the closure of CNG channels, reduction in Ca^2+^ influx/[Ca^2+^]_i_, and the subsequent activation of the GCAP/RetGC complex (Figure 1). Thus, CNG channels not only play a pivotal role in phototransduction but also regulate cellular cGMP levels.

## 3. Dysregulation of cGMP Signaling in Photoreceptor Degeneration

Among the various cellular alterations that occur in photoreceptor degeneration and the potential causative factors, dysregulation of cGMP signaling represents one of the top pathogeneses. The dysregulation is primarily manifested as the abnormally elevated cellular cGMP levels and the subsequent elevation of PKG signaling, which alters a wide range of cellular activities, resulting in impaired cellular homeostasis, cellular stress, and cell death [14,49]. The accumulation of cGMP also leads to an increase in Ca^2+^ influx/Ca^2+^ overload via CNG channels, as well as the subsequent harmful outcomes. Normally, only a small fraction of the total CNG channels is open by cGMP in dark-adapted photoreceptors, and the elevated cGMP leads to opening an excessive number of the channels in the dark, thus abnormally increasing the influx of Ca^2+^ and Na^+^. The defects/mutations in various molecules involved in the regulation of cGMP production/degradation, including RetGC1, RD3, GCAP1, PDE6, and CNG channels, are the primary causes of the dysregulation of cGMP signaling in photoreceptors.

### 3.1. Dysregulation of RetGC1, RD3, and GCAP1

There are over 100 mutations in *GUCY2D* (encoding RetGC1) identified to date. These mutations are mainly associated with autosomal-dominant CRD (adCRD) [50,51,52,53,54,55] and autosomal-recessive LCA (arLCA) [56,57,58,59,60]. The dominant, gain-of-function mutations in *GUCY2D* are associated with the degeneration of both rods and cones, causing adCRD, whereas the *GUCY2D* LCA mutation (arLCA) primarily results in the dysfunction of signaling, leading to the loss of photoreceptor function/blindness [61]. Mutations in *GUCY2D* account for about 40% of the total adCRD cases and 10–20% of the total arLCA cases [53]. The adCRD mutations are all missense variants, and the mutant proteins are functional. Studies using animal models and cell culture models show that many of these mutations enhance the sensitivity of RetGC1 to GCAP, resulting in excessive cGMP production and enhanced Ca^2+^ influx [55,62,63,64]. The residue Arg^838^, located in the dimerization domain of RetGC1, is a mutation hotspot for adCRD, and it is the typical example of overactivation dysregulation. The phenotype in R838S RetGC1 transgenic mice is completely prevented by the deletion of GCAP [63]. In contrast, the LCA phenotype is associated with biallelic null mutations in which the RetGC1 activity is severely reduced or absent due to impaired sensitivity to GCAPs [54,56,57], thereby leading to less production of cGMP and dysfunction of signaling. Of note, the *GUCY2D* LCA mutations do not necessarily result in the degeneration of photoreceptors. Although *GUCY2D* arLCA patients display retinal blindness from birth, the vast majority of photoreceptors remain alive throughout adulthood. It was recently shown that *GUCY2D* replacement by gene therapy quickly improves cone and rod sensitivity after decades of blindness [65,66]. Other, less common phenotypes associated with mutations in *GUCY2D* include autosomal recessive CRD (arCRD) [67] and autosomal recessive congenital stationary night blindness (arCSNB) [68]. It should be noted that the *GUCY2D* arCSNB patients show some age-related rod loss in the peripheral retina [68]. In addition, the deficiency of *RD3* causes severe photoreceptor degeneration and blindness in recessive LCA, as well as in the *rd3* mouse strain [69,70]. This is because a deficiency of the RD3 protein causes the aberrant activation of cGMP production, via the RetGC/GCAP complex in the inner segment, triggering the cell death process.

There are about 20 mutations in *GUCA1A* (encoding GCAP1) identified to date. These mutations are mainly associated with cone degenerations, including cone dystrophy, cone–rod dystrophy, and macular dystrophy [71,72,73,74,75,76,77]. Studies using animal models and cell culture models show that these mutations, such as the Y99C and G86R mutations [78,79], reduce the sensitivity of GCAP to Ca^2+^ and subsequently increase RetGC1 activity, leading to the overproduction of cGMP, enhanced CNG channel activity, and enhanced Ca^2+^ influx [71,72,74,78,79].

### 3.2. Deficiency of PDE6

Mutations in *PDE6A* and *PDE6B* are associated with RP and adCSNB [80,81,82,83,84], whereas mutations in *PDE6C* and *PDE6H* are associated with cone dystrophy and achromatopsia [85,86,87,88]. These mutations are all loss-of-function mutations, leading to the cellular accumulation of cGMP and increased Ca^2+^ influx in photoreceptors. Mouse models with defects of *Pde6* genes have been widely studied to understand the disease pathogenesis. The *rd1* mouse has a non-sense mutation in the cGMP-binding domain of *Pde6b*, and as a result, the mRNA/protein is not produced. These mice do not show detectable PDE6 activity [89,90], and they exhibit dramatic increases in cellular cGMP levels between postnatal day 10 (P10) and P14 [91]. The *rd1* mice display disorganized outer segments at P7 and the complete loss of rods by about P21, followed by secondary cone degeneration [92,93]. The *rd10* mouse harbors an autosomal missense R560C mutation in *Pde6b*, proceeding with slower rod degeneration compared to *rd1* mouse. The *Rd10* retinas appear nearly normal for the first 2 weeks, but then, they rapidly deteriorate with a severe loss of photoreceptors [94,95,96]. The mutation does not alter the production of *Pde6b* mRNA, but it dramatically reduces maximal/basal activity of PDE6, leading to the accumulation of cellular cGMP [97]. The *Cpfl1* [cone photoreceptor function loss (1)] mouse harbors a 116-bp insertion between exon 4 and 5, as well as an additional 1-bp deletion in exon 7 in the *Pde6c* gene, leading to an in-frame shift, introducing premature termination codons, the loss of activity of the enzyme, and the accumulation of cGMP [49,88,98]. *Cpfl1* mice display the early-onset, rapid/severe cone degeneration phenotype, including the loss of cone function and death of cones [88,98,99,100].

As mentioned above, the disease-causing mutations in *Pde6* genes are all loss-of-function mutations, leading to the cellular accumulation of cGMP and increased Ca^2+^ influx in photoreceptors. Of note, it seems that the cGMP production feedback via the GCAP/RetGC complex, subsequent to increased [Ca^2+^]_i_, cannot decrease the intracellular levels of cGMP in *Pde6* mutants. The cellular mechanism(s) underlying this observation is unknown at this time. The observation may suggest that the feedback regulation by the GCAP/RetGC complex is less sensitive to the elevation of [Ca^2+^]_i_ than to the reduction in [Ca^2+^]_i_. It may also imply that the feedback regulation is blunted, for some reason, in the absence of functional PDE6. Nevertheless, the feedback regulation of cGMP production by the GCAP/RetGC complex, in the absence of functional PDE6, merits further investigation.

### 3.3. Deficiency of CNG Channel

Mutations in CNG channels are associated with photoreceptor degeneration. Mutations in *CNGA1* and *CNGB1* are associated with arRP, accounting for 2–3% of arRP cases [101,102,103,104]. To date, there are about 40 mutations identified in *CNGA1* [105] and over 80 mutations identified in *CNGB1* [106]. Most of these mutations are loss-of-function mutations. Mutations in *CNGA3* and *CNGB3* are associated with achromatopsia, progressive CRD, and early-onset macular degeneration [107,108,109]. There are over 100 and 40 mutations identified in *CNGA3* and *CNGB3*, respectively, accounting for 70–80% of the total achromatopsia cases [108,110,111]. Mouse models with CNG channel deficiency mimic phenotypes in human patients, and they are manifested as reduced photoreceptor function and retinal degeneration [112,113,114]. Reduced [Ca^2+^]_i_ and accumulation of cellular cGMP have been shown in the cones of CNG channel-deficient mice [113,114,115,116]. The cellular cGMP level increased at P8, peaked around P10–P15, remained high from P30–P60, and returned near the control level at P90 [113]. The abnormal accumulation of cGMP in photoreceptors is also demonstrated in mice with *Cngb1* deficiency [117]. Unlike that in PDE6 deficiency, in which the impaired degradation of cGMP leads to its accumulation, CNG channel deficiency results in a reduction in [Ca^2+^]_i_, which subsequently leads to the overproduction of cGMP via the Ca^2+^/Mg^2+^-GCAP/RetGC complex.

### 3.4. Deficiency of Other Photoreceptor Specific Proteins

Interestingly, the accumulation of cGMP has been observed in *rd2/rds* mice with deficiencies of peripherin/Rds [49,118]. Peripherin/Rds is a disc membrane-integral protein. Dysfunction of this protein leads to the impaired morphogenesis of the outer segments, and it is associated with RP and macular degeneration [119,120]. How the dysfunction of peripherin/Rds leads to the accumulation of cGMP remains unknown currently. It might be related to the role of structural integrity of the outer segments in the biosynthesis/degradation of cGMP in photoreceptors.

## 4. Experimental Evidence Supporting the Contribution of Elevated cGMP Signaling to Photoreceptor Degeneration

Evidence supporting the contribution of the elevated cGMP signaling to photoreceptor degeneration is mainly obtained from the studies by depleting cGMP, deleting a CNG channel, or inhibiting PKG. All these approaches lead to photoreceptor protection in mouse models with the dysregulation of cGMP signaling.

### 4.1. Depletion of cGMP Reduces Photoreceptor Degeneration

The depletion of cellular cGMP reduces photoreceptor degeneration in mouse models with PDE6 deficiency and CNG channel deficiency. Knockdown of *Gucy2e* increases visual function and photoreceptor survival in *Pde6b* mutant mice [121]. The deletion of *Gucy2e* rescues the retinal phenotype in *Cnga3^−/−^* and *Cnga3^−/−^/Nrl^−/−^* mice (*Cnga3*-deficient mice on a cone dominant *Nrl^−/−^* background), manifested as increased cone density and with expression levels of cone-specific proteins [113,122,123]. The suppression of guanosine nucleotides/cGMP production, using mycophenolate mofetil (MMF), protects photoreceptors in *rd10* and *rd1* mice. MMF treatment significantly delays the onset of retinal degeneration, cGMP-dependent photoreceptor cytotoxicity, retinal/visual function, and microglial activation in *rd10* and *rd1* mice [124]. In addition, the overexpression of RD3 attenuates photoreceptor degeneration in transgenic mice expressing the human R838S RetGC1 dominant mutant [125]. The R838S RetGC1 mutation increases the enzyme’s affinity for Mg^2+^-GCAP (the activated form of GCAP) or makes it more difficult for RD3 to prevent the aberrant activation of RetGC by GCAP in the inner segment, leading to the increased synthesis of cGMP. The overexpression of RD3 leads to the suppression of RetGC1, as well as the rescue of the retinal phenotype observed in R838S RetGC1 mutant mice.

### 4.2. Inhibition of CNG Channel to Decrease Ca^2+^ Influx Reduces Photoreceptor Degeneration

The inhibition/deficiency of a CNG channel reduces retinal phenotypes in PDE6-deficient mice. Knockdown of *Cnga1* increases visual function and photoreceptor survival in *Pde6b* mutant mice [121]. Similarly, the deletion of *Cngb1* reduces photoreceptor death in *Pde6g* mutant mice [117]. Furthermore, the deletion of *Cnga3* reduces cone degeneration in *cpfl1* mice [126]. It should be pointed out that the cGMP level remains pathologically high in PDE6-deficient mice after the deletion of the CNG channel. However, photoreceptor viability and outer segment morphology are greatly improved, suggesting that deleterious Ca^2+^ influx is the main cause of the rapid death of photoreceptors [127].

### 4.3. Inhibition of PKG Reduces Photoreceptor Degeneration

The sole enzyme targets of cGMP are the PKGs. PKGs are serine/threonine kinases that are present in a variety of eukaryotes. There are two genes, *Prkg1* and *Prkg2,* encoding for PKGs. The *Prkg1* encodes for PKGI, whereas the *Prkg2* encodes for PKGII [13,128,129]. The expression and activity of PKG are upregulated in the retinas of mice with abnormally elevated cellular cGMP levels [113,114,122]. In line with this, the inhibition of PKG reduces photoreceptor degeneration in mouse models with the dysregulation of cGMP signaling. Treatment with PKG inhibitors/cGMP analogues, including the classic compounds KT5823 and (*R*_P_)-8-Br-cGMPS, as well as the newly developed compounds CN03 and CN04, protects photoreceptors in PDE6-deficient mice, rd2/*rds* mice, and CNG channel-deficient mice [49,122,130]. The contribution of PKG to photoreceptor degeneration is also demonstrated by the genetic deletion of the enzymes. The deletion of *Prkg2* reduces ER stress and cone death, resulting in long-lasting preservation for the morphology and structure of cone photoreceptors in *Cnga3*-deficient mice [131]. Additionally, the deletion of *Prkg1* reduces rod degeneration in *Cngb1*-deficient mice [117].

## 5. The Cellular and Molecular Mechanisms Underlying cGMP Signaling-Induced Photoreceptor Degeneration

**Figure 2:**
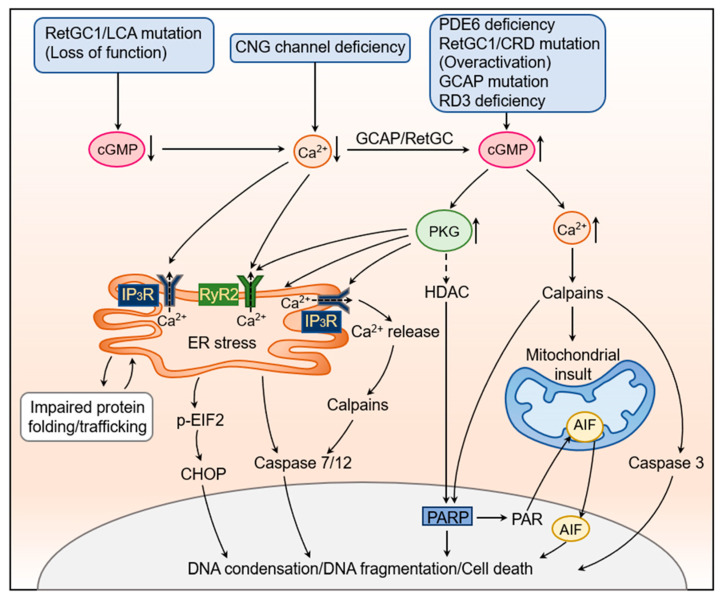
The cellular and molecular mechanisms underlying cGMP signaling-induced photoreceptor degeneration. Overactivation of RetGC/GCAP or deficiency of PDE6 leads to the accumulation of cGMP and the subsequent intracellular Ca^2+^ overload. Deficiency of RD3 leads to the aberrant activation of the RetGC/GCAP complex in the inner segment, leading to excessive cGMP production. Deficiency of the CNG channel leads to a reduced intracellular Ca^2+^ level and the subsequent accumulation of cGMP via the Ca^2+^/Mg^2+^-GCAP/RetGC complex. The deficiency of RetGC/GCAP leads to reduced cellular cGMP and [Ca^2+^]_i_. The elevated cGMP/PKG signaling and impaired Ca^2+^ homeostasis lead to cellular stress/death. The elevated cGMP/PKG signaling induces ER stress, leading to cell death via the activation of CHOP and caspase-7/12. Along with a reduced cellular Ca^2+^ level, it causes ER Ca^2+^ dysregulation by activating the IP_3_R1 and RyR2 channels and promoting the release of Ca^2+^ from the ER. The activated cGMP/PKG signaling may also induce HDAC and PARP, leading to cellular stress/cell death, directly and via the release of AIF from the mitochondria. The elevated cGMP/Ca^2+^/calpain signaling induces cellular stress/death by activating caspases, releasing AIF from the mitochondria, and stimulating PARP.

There are multiple mechanisms involved in cGMP dysregulation-induced photoreceptor degeneration (Figure 2). The elevated cGMP/PKG signaling impairs endoplasmic reticulum (ER) Ca^2+^ homeostasis and induces ER stress-associated cell death. Similarly, the low [Ca^2+^]_i_ in CNG channel deficiency harms the ER Ca^2+^ homeostasis and deteriorates ER function. The elevated cGMP/PKG signaling also activates histone deacetylase (HDAC)/poly-ADP-ribose polymerase (PARP) signaling, leading to DNA condensation/damage and cell death. The elevated cGMP signaling and the subsequent cellular Ca^2+^ overload triggers the calpain-associated cell death pathway. Moreover, the ER stress, activation of PARP signaling, and activation of calpain signaling induce the mitochondrial insult and release of the apoptosis-inducing factor (AIF) to worsen the death process.

### 5.1. Elevated PKG Signaling

#### 5.1.1. Excessive PKG Signaling Induces ER Stress

The ER is a large membrane-enclosed cellular organelle dedicated to biosynthesis, folding, and the assembly of membrane/secretory proteins, and it functions as a free Ca^2+^ storage. ER homeostasis is critical for its function, and impaired ER homeostasis will lead to unfolded protein response (UPR)/ER stress, impaired protein processing/trafficking, and impaired ER-associated protein degradation (ERAD). Under the stress conditions or accumulation of incorrectly folded/unfolded proteins, ER will undergo UPR/ER stress, which triggers cytotoxicity and cell death. ER stress is typically characterized by the activation of the three pathways/arms, including the pancreatic ER kinase (PKR)-like ER kinase (PERK)/eukaryotic-initiation factors 2α (phospho-eIF2α) pathway, the cleaved activating transcription factor 6 (ATF6) pathway, and the inositol-requiring kinase 1 (IRE1) pathway [132,133,134]. There are a variety of stress factors, originating from both cytosol and ER lumen, activating the three arms and initiating UPR, ER stress, and, ultimately, cell death.

Elevated PKG signaling impairs ER homeostasis and induces ER stress-associated photoreceptor death (Figure 2). This cellular event is well characterized in mice with CNG channel deficiency. Mice with cone CNG channel deficiency show ER stress-associated cone death, manifested as increased ER stress marker proteins, including Grp78/Bip, phospho-eIF2, phospho-IP_3_R, and CCAAT/enhancer-binding protein homologous protein (CHOP), along with impaired protein trafficking and impaired ERAD [114,122,123,135]. ER stress-associated cell death in CNG channel deficiency is also manifested by the up-regulation of the cysteine protease calpains and the cleavage of the cysteine proteases, caspase-12 and caspase-7. Caspase-12 is predominantly localized at the ER, activated upon ER stress and Ca^2+^ release from the ER store, and subsequently translocated into the nucleus to induce apoptosis [136,137]. Caspase-7 resides in the ER neighborhood along with caspase-12 [138]. It is activated under ER stress, and the activated caspase-7 cleaves procaspase-12 to generate active forms of the protease [138]. The involvement of caspase-12, and its translocation to the nucleus, is also demonstrated in *Pde6b* mutant retinas [139]. Treatment with a chemical chaperone (tauroursodeoxycholic acid, TUDCA) or a molecular chaperone (11-*cis*-retinal) reduces ER stress responses/cone death in CNG channel deficiency and improves protein trafficking/outer segment location [123,135], demonstrating a role of ER stress in the photoreceptor degeneration. The link between activated PKG signaling and ER stress/cell death in CNG channel deficiency is demonstrated by the depletion of cGMP/the deletion of RetGC1 and inhibition of PKG using PKG inhibitors and PKG deletions. The depletion of cGMP or inhibition of PKG significantly reduces ER stress/cone death and improves protein trafficking to the outer segments in CNG channel-deficient mice [113,123,135].

#### 5.1.2. Excessive PKG Signaling Impairs ER Ca^2+^ Homeostasis

As a storage of free Ca^2+^, ER plays a pivotal role in cellular/ER Ca^2+^ homeostasis. ER Ca^2+^ homeostasis and regulation are vital for ER to function properly, including protein folding/trafficking. ER Ca^2+^ homeostasis is governed by the ER Ca^2+^ channels, inositol 1,4,5-trisphosphate receptors (IP_3_Rs) and ryanodine receptors (RyRs), on the ER membrane, for efflux out of the ER into cytosol, and the sarco/endoplasmic reticulum Ca^2+^-ATPase (SERCA) for influx into the ER. There are three IP_3_R isoforms: IP_3_R1, IP_3_R2, and IP_3_R3. In the mouse retina, IP_3_R1 is the major IP_3_R isoform, its mRNA level is approximately 6 to 10-fold higher than the mRNA level of IP_3_R3, whereas IP_3_R2 mRNA is not detectable in transcript expression studies [123]. There are three RyR isoforms: RyR1, RyR2, and RyR3 [140]. All three isoforms of RyRs are expressed in photoreceptors, and RyR2 is identified as the major form in the photoreceptors [140]. The activities of ER Ca^2+^ channels are highly regulated by their respective ligands, IP_3_ and ryanodine, cytosolic Ca^2+^ levels, and other regulatory signaling, including PKG signaling.

The impairment of ER Ca^2+^ homeostasis in the dysregulation of PKG signaling is well characterized in mice with CNG channel deficiency (Figure 2). The expression/activity of IP_3_R1 and RyR2 is significantly increased in these mice, along with protein mistrafficking/mislocalization, ER stress, and impaired ERAD [123]. The inhibition of PKG by chemical inhibitors or genetic deletion, or the depletion of cGMP by *Gucy2e* deletion, reduces the expression/activity of IP_3_R1 and RyR2, along with reduced ER stress/cone death and improved protein trafficking/outer segment localization [122,131,141,142]. The regulation of PKG on the ER calcium channels is also shown in a cell culture model system. Treatment with the cGMP analogue significantly enhances mRNA levels of IP_3_R1 and RYR2 in cultured photoreceptor-derived Weri-Rb1 cells [141]. Furthermore, the inhibition of IP_3_R1 and RyR2, by chemical inhibitors or genetic deletion, reduces ER stress/photoreceptor death and improves protein trafficking/outer segment localization and ERAD [123,142,143]. Thus, the elevated PKG signaling stimulates ER Ca^2+^ channels, leading to increased ER Ca^2+^ release/depletion and impaired ER function/processing.

#### 5.1.3. Other Potential Targets of PKG in Photoreceptor Degeneration

As a serine–threonine kinase, PKGs have numerous phosphorylation protein targets. Although the regulation of PKG signaling in ER stress/ER Ca^2+^ homeostasis has been characterized in CNG channel deficiency, the substrates of PKG and the downstream pathways triggered by its activation in retinal degeneration remain not fully understood. A recent kinome activity-profiling study, which applies multiplex peptide microarrays to identify proteins whose phosphorylation are significantly altered by PKG activation, has identified several potential PKG target proteins in *rd1* mouse retinal explants [144]. Among the PKG substrates are potassium channels belonging to the Kv1 family (KCNA3, KCNA6), cyclic AMP-responsive element-binding protein 1 (CREB1), DNA topoisomerase 2-α (TOP2A), 6-phosphofructo-2-kinase/fructose-2,6-biphosphatase 3 (F263), and the glutamate ionotropic receptor kainate 2 (GRIK2). The retinal expression of these PKG targets is confirmed by immunofluorescence labeling [144]. The elevated CREB signaling is also observed in CNG channel-deficient retina, and the deletion of *Gucy2e* reverses this alteration [141]. In addition, the transcriptome analysis of *rd1* retinal explants suggests that PKG signaling negatively affects oxidative phosphorylation and mitochondrial function [145]. However, whether these molecules contribute to photoreceptor degeneration remains to be investigated.

### 5.2. Impaired Cellular Ca^2+^ Homeostasis

#### 5.2.1. Ca^2+^ Overload and Calpain Activation

In PDE6 deficiency or overactivation of RetGC1, the accumulation of cGMP will lead to a constitutive opening of the CNG channel and intracellular Ca^2+^ overload (Figure 2). The most prominent consequence of Ca^2+^ overload is the activation of the calcium-activated cysteine protease calpains. Calpains exist as inactive proenzymes in the cytosol. When the intracellular Ca^2+^ level is high, it triggers the conversion of the proenzymes to their active forms. Activated calpains then cleave a variety of cytoplasmic and nuclear substrates, including caspase 3 and PARP [146,147]. The activation of calpains also induces the release of AIF from mitochondria, leading to prolonged proteolysis and apoptosis/necroptosis [114,139,148,149,150,151,152]. Retinas of *rd1* and *rd10* mice show increased calpain activity, which peaks synchronously with cell death [139,148,150,152]. An elevation of calpain activity is also observed in *Cpfl1* mice [98]. The contribution of calpains to cell death in retinal degeneration is demonstrated by using the calpain-specific inhibitors. Treatment with calpain inhibitor XI significantly reduces cell death in *rd1* and *rd10* mice [148,149]. Interestingly, the contribution of calpain activity to photoreceptor death is also shown in an induced animal model of retinal degeneration. The activation of calpains and the calpain-specific proteolysis of α-spectrin are observed in experimental rats treated with *N*-methyl-*N*-nitrosourea (MNU), an alkylating agent that interacts with DNA, specifically targets retinal photoreceptors, and causes oxidative stress/damage. The administration of a calpain inhibitor induces a significant protective effect against photoreceptor loss in MNU-treated rats [153].

#### 5.2.2. Ca^2+^ Depletion

Ca^2+^ homeostasis is crucial for normal cell function. Ca^2+^ overload is cytotoxic. Likewise, Ca^2+^ depletion also renders harmful effects. Reduced [Ca^2+^]_i_ has been shown in the cones of CNG channel-deficient mice [123]. Similar pathophysiological conditions may be present in LCA-RetGC mutation/RetGC1 deficiency [19,61,154], as well as in a *rd3* mutant mouse [155]. When the cytosolic Ca^2+^ level is low/reduced, it will affect not only the function of many Ca^2+^ binding/regulating proteins but also the function/homeostasis of ER and mitochondria. More profoundly, when the CNG channel is intact, the reduced cellular Ca^2+^ level will activate GCAP/RetGC, leading to the accumulation of cGMP and subsequent activation of PKG (Figure 2).

### 5.3. Other Factors

#### 5.3.1. HDAC and PARP

HDACs are enzymes that catalyze the removal of acetyl functional groups from the lysine residues of both histone and nonhistone proteins. Excessive activation of HDAC is associated with photoreceptor death in mouse models with cGMP signaling perturbation (Figure 2). HDAC activity is increased in the retinas of *rd1* and *Cpfl1* mice, and treatment with HDAC inhibitors reduces photoreceptor degeneration in these mice [156,157]. Nevertheless, how HDAC is activated in the retinas of these mice remains less understood. Based on the comparative localization studies [158], PKG might be the upstream activator. PARP is a DNA-repairing enzyme that is involved in genomic stability and programmed cell death through the formation of poly (ADP-ribose) polymers (PAR), and its activation causes a parthanatos-related form of cell death [159]. *Rd1* photoreceptors actively undergoing cell death show a great elevation of PARP activity. The elevated PARP is co-labeled with oxidatively damaged DNA and the nuclear translocation of AIF. Moreover, treatment with the PARP inhibitor reduces cell death in *rd1* retinal explants [160]. The association of PARP activation with HDAC is demonstrated by using HDAC inhibitors. Treatment with HDAC inhibitors reduces PARP activation and photoreceptor degeneration in *rd1* mice [156]. In addition to HDAC, PARP activation may result from elevated cellular Ca^2+^ and the activation of calpain [161,162]. PARP is cleaved, by purified calpain, into fragments, suggesting it might be one of the calpain substrates [147].

#### 5.3.2. AIF

AIF is the protein that triggers chromatin condensation and DNA degradation. It resides, normally, within the intermembrane space of mitochondria. Upon certain death stimuli/mitochondrial damage, AIF translocates into the cytosol and, ultimately, the nucleus, where it contributes to DNA fragmentation and chromatin condensation, initiating a caspase-independent cell death process [163,164]. Nuclear translocation of AIF is observed in the retinas of CNG channel-deficient mice [114] and *rd1* mice [160], suggesting a potential role of mitochondrial insult and nuclear translocation of AIF in retinal degeneration with the dysregulation of cGMP signaling. The activation of calpain and PARP has been shown to be associated with the activation of AIF [165,166,167]. In *rd1* mice, the nuclear translocation of AIF is overlapped with PARP activity staining [160], suggesting that PARP mediates the translocation of AIF. Furthermore, calpain inhibitors interfere with the AIF activation/nuclear translocation in the *rd1* retina [139], supporting an involvement of calpain in the activation of AIF.

## 6. Summary and Perspectives

Photoreceptor cGMP level is tightly controlled by a well-developed, feedback regulatory system. The Ca^2+^/Mg^2+^-GCAP/RetGC complex, with the modulation of RD3, governs the biogeneration of cGMP, whereas PDE6 is the sole force for degradation of cGMP. The binding of cGMP to the CNG channel regulates cellular Ca^2+^ levels, membrane potential, and phototransduction. As second messengers, cGMP and Ca^2+^ regulate a variety of cellular activities via their downstream targets. The dysregulation of cellular cGMP homeostasis leads to impaired phototransduction and photoreceptor degeneration, and it is associated with retinal diseases, including RP, LCA, CRD, and achromatopsia. Overactivation of RetGC/GCAP, deficiency of RD3, or deficiency of PDE6 lead to an accumulation of cGMP. The deficiency of a CNG channel leads to the accumulation of cGMP via the Ca^2+^/Mg^2+^-GCAP/RetGC complex. The deficiency of a CNG channel reduces Ca^2+^ influx, leading to reduced [Ca^2+^]_i_ and the subsequent activation of RetGC. Activated cGMP/PKG signaling induces ER stress and the impairment of ER Ca^2+^ homeostasis. Either cellular Ca^2+^ overload or Ca^2+^ depletion will impair cellular homeostasis, leading to cell stress/death. In PDE6 deficiency, RD3 deficiency, or RetGC overaction, the activated cGMP/PKG signaling, along with Ca^2+^ overload/calpain activation, plays a significant role in the disease pathogenesis and cell death progression. In CNG channel deficiency or RetGC deficiency, the activated cGMP/PKG signaling, along with cellular Ca^2+^ depletion, plays a significant role in the disease pathogenesis and cell death progression. The activated cGMP/PKG signaling may also induce HDAC and PARP, leading to cell stress/death directly and via mitochondrial dysfunction/the release of AIF. Thus, targeting cGMP/PKG signaling in photoreceptors and the downstream target cellular organelles/molecules represents a significant approach to slow photoreceptor death in retinal degenerative diseases.
